# Radiographic comparison of cervical spine motion using LMA Fastrach, LMA CTrach, and the Macintosh laryngoscope

**DOI:** 10.3906/sag-1906-135

**Published:** 2019-12-16

**Authors:** Gözde İNAN, Nurdan BEDİRLİ, Zerrin ÖZKÖSE ŞATIRLAR

**Affiliations:** 1 Department of Anesthesiology and Reanimation, Faculty of Medicine, Gazi University, Ankara Turkey

**Keywords:** Neuroanesthesia, spinal surgery, intubation, spine imaging

## Abstract

**Background/aim:**

The optimal technique for airway management in patients with cervical pathology remains unclear. Intubating laryngeal mask airway devices such as LMA CTrach and LMA Fastrach have not been compared for cervical spine (C-spine) movements in the context of cervical pathology. The present study aimed to determine upper C-spine movements by radiography during intubation with different devices as well as comparing the duration and success of intubation in cervical surgery.

**Materials and methods:**

Sixty patients scheduled for elective cervical surgery were registered in this prospective, randomized study. Patients with cervical trauma/injury, previous neck surgery, and body mass index (BMI) of >35 kg/m^2^ were excluded. Participants were randomized to one of the 3 groups: LMA CTrach, LMA Fastrach, or the Macintosh laryngoscope. C-spine motion was evaluated by measuring angles created by bordering vertebrae at cervical 1/2 and 2/3 (C1/2, C2/3) segments on 2 lateral cervical radiographs for each patient. Intubation time, ease of intubation, number of attempts, and success rate were also documented.

**Results:**

Demographic data were similar in all the groups. The cervical movement with LMA CTrach and LMA Fastrach compared to the Macintosh laryngoscope were similar at C1/2. However, LMA CTrach significantly reduced extension compared to LMA Fastrach and Macintosh laryngoscopes at C2/3. Duration of intubation was significantly shorter with the Macintosh laryngoscope. The rate of successful intubation was 80% with LMA Fastrach and 100% with both LMA CTrach and the Macintosh laryngoscopes.

**Conclusion:**

The LMA CTrach laryngoscopy involves less upper C-spine movement than the LMA Fastrach and does not increase the duration of the intubation period.

## 1. Introduction

Intubation with various airway devices causes cervical spine (C-spine) extension to some degree. The process gains importance especially in emergency situations with cervical injury and in C-spine surgeries [1,2]. The main concerns of anesthesiologists for airway management both in cervical injury and C-spine surgeries include avoiding prolonged intubation time and preventing neurologic damage due to excess cervical movements [2,3].

Conventional laryngoscopy with a Macintosh blade remains the most familiar way to enable tracheal intubation. However, maneuvering for intubation and adjustment of the oropharyngeal and laryngeal axes produces C-spine movement [4]. In a recent systemic review, alternative intubation techniques performed in patients with cervical immobilization were compared with the Macintosh laryngoscopy [5]. The authors concluded that evidence of the efficacy of alternative devices was missing. Fiberoptic intubation is still the most ideal technique to secure an airway in patients with predicted difficult intubation [2]. Fiberoptic laryngoscopy is considered to facilitate the least cervical movement during laryngoscopy but has several limitations like requiring a cooperative patient and lasting a long time, making it unsuitable for emergencies [2,6]. However, as intubating laryngeal mask airways (ILMAs), LMA CTrach and LMA Fastrach are alternative techniques that may be useful when fiberoptic bronchoscope is not available. ILMAs have been validated for ventilation and as a conduit to tracheal intubation in patients with difficult airways. LMA CTrach was developed from LMA Fastrach with additional advantageous features like visualizing the glottis and intubation process [7,8]. However, both devices require experience to operate and the administration may prolong intubation time. 

Previous radiographic and fluoroscopic studies, purporting to evaluate C-spine movement during intubation with various intubating techniques, were carried out. [4,6,9–12]. Intubating laryngeal mask airways have been compared for the success of tracheal intubation [13,14]. However, there have been no studies investigating C-spine movements using both ILMAs. Moreover, the effects of different intubation techniques on cervical movements were followed among healthy subjects in nonemergency situations not including neck, throat, or cervical surgeries in patients with cervical immobility due to manual in-line stabilization or cervical collars and in cadaveric models in most studies [3,12]. Comparative studies performed on patients with cervical pathologies who undergo cervical surgery are lacking in the literature. Therefore, this prospective, randomized radiographic study was conducted to compare the movements of the upper C-spine (C1, C2, and C3) during laryngoscopy via LMA Fastrach, LMA CTrach, and Macintosh laryngoscopes in patients with lower cervical pathology undergoing C-spine surgery. The secondary outcomes were the comparison of the intubation success and the duration. 

## 2. Materials and methods

The ethics committee of the School of Medicine of Erciyes University approved this prospective, randomized, and controlled study (reference number: 2011/177). All the patients were informed about the study and written consent was obtained. Inclusion criteria were American Society of Anesthesiologists (ASA) physical status of I–III, ages between 18 and 70 years old, and patients undergoing elective C-spine surgery. Patients with documented cervical trauma or injury, previous neck surgery, body mass index (BMI) of >35 kg/m2, the possibility of pregnancy, or failed tracheal intubation (more than 2 intubation attempts with a device) were excluded. All patients’ preoperative height, weight, ASA physical status, BMI, and Mallampati scores were documented. 

Electrocardiography, noninvasive blood pressure measurement, and pulse-oximeter were monitored in all patients in a standard fashion. Before anesthesia induction, the patients were positioned in a neutral position. Following preoxygenation, anesthesia was induced with intravenous (IV) 2 mg/kg propofol, 1 mg/kg lidocaine, 0.5 µg/kg remifentanil, and 0.6 mg/kg rocuronium, and maintenance was achieved with sevoflurane in an air-oxygen mixture and IV infusion of remifentanil. 

In the operating room, 63 patients were randomly assigned by a computer random number generator to one of the 3 groups, corresponding to the 3 airway devices: Macintosh laryngoscope (Group M), LMA Fastrach (Group F), or LMA CTrach (Group C). We did not stabilize the head and neck or apply cricoid pressure. In Group M patients, conventional direct laryngoscopy was performed. The diameter of the endotracheal tube (ETT) was 7 or 7.5 mm in females and 8 mm in males. In Group F, size 3, 4, or 5 LMA Fastrach (LMA North America Inc., San Diego, CA, USA) depending on the weight of the patient was applied in accordance with the manufacturer’s instructions. A silicone reinforced ETT was lubricated and inserted. If the right position of the tube was achieved without resistance, the ETT was advanced into the trachea and the cuff was inflated. The position of the ETT was determined by auscultation and capnography. Subsequently, a special stabilizing rod was used to remove the LMA Fastrach. In Group C, size 3, 4, or 5 LMA CTrach (LMA North America Inc., San Diego, CA, USA) was preferred depending on the weight of the patient. Initially, LMA CTrach was inserted without the viewer on, just as LMA Fastrach was applied. The cuff was inflated and the patient was ventilated. The viewer was then attached to the connector while holding the handle. When a clear image of the glottis and vocal cords was achieved, an ETT was inserted and intubation was visualized, and the ETT cuff was inflated. The viewer was then detached, and LMA CTrach was removed following the same procedure as for LMA Fastrach. The same experienced anesthesiologist, who had previously performed at least 100 intubations with each of LMA CTrach and LMA Fastrach and 500 intubations with the Macintosh laryngoscope, performed all laryngoscopies in order to minimize interoperator variability. 

The intubation techniques were recorded with a portable X-ray machine. Two recordings were performed a steady distance from the patient and the tube in the lateral position. The first was taken in a neutral position before the intubation process and the second was taken when the best view of the glottis was achieved with the LMA CTrach and Macintosh laryngoscopes. For the LMA Fastrach, if no resistance was felt as the ETT was advanced through the mask aperture into the trachea, it was thought to be at correct tube positioning and indicated time for the fluoroscopy. The movements of the cervical 1-2 and 2-3 segments were evaluated in radiographs. First we drew a reference line, which follows the cervical 2 dorsal alignment. Then we drew two more lines, which transected the driven reference line, the first one between the anterior and posterior arches of the cervical 1 and the other one through the cervical 3 basal plate of C3. Thus, there were two angles observable: one between the reference line and the arches of cervical 1, named alpha (α), while the second angle, named beta (β), was located between the reference line and the line passing through the cervical 3 basal plate. The lines were drawn and an investigator, who was unaware of the study group assignments, randomly measured angles using a goniometer in degree (°) unit. 

The duration of intubation, the number of attempts, and the intubation success rate were recorded. The duration of the intubation was recorded between the passage of the intubation device through the lips and inflation of the tracheal cuff. Intubation was considered successful if the patient was intubated in less than two attempts and failed in case of more than two. 

Estimated from the data of Watts et al. [15] (12.9 ± 2.1° extension) and based on the assumption of α = 0.05, β = 0.8 to detect 15% reduction in the movement of the upper C-spine, each group would need to include at least 18 patients. Therefore 20 patients per group were planned to be enrolled due to the probability of a 10% drop-out rate. SPSS 15.0 for Windows (SPSS Inc., Chicago, IL, USA) was used for the statistical analysis. Data are expressed as mean ± standard deviation (mean ± SD), median (min–max), or numbers (n) and percentages (%). Discrete variables (sex, ASA classification, Mallampati scores, success rate) were compared using the chi-square test. For numeric parameters of between-group comparisons, one-way analysis of variance (in the case of parametric test conditions) or the Kruskal–Wallis tests (if parametric test conditions could not be obtained) were used. Multiple comparisons of the Kruskal–Wallis test were done by Mann–Whitney U test with Bonferroni correction. Analysis of variance was used for repeated measures of arterial pressure, heart rate, and oxygen saturation and the angle measurements for both between-group and intergroup comparisons. P < 0.05 was considered statistically significant.

## 3. Results

Sixty-three patients were allocated to the intervention groups. For one patient, intubation with LMA Fastrach was not possible despite adequate manipulation, even after the third attempt. Following 3 esophageal intubations, correct intubation was achieved by direct laryngoscopy and this patient was excluded from the study. In Group C, the vocal cords of 1 patient could not be visualized despite all maneuvers and the patient was also excluded. One of the patients’ radiographic images could not be printed out so the angle measurement could not be analyzed in Group M. Subsequently, 20 patients per group were analyzed.

The demographic data, Mallampati scores, and ASA physical status classification (P > 0.05) were similar in all groups (Table 1). The hemodynamic and ventilation parameters, such as arterial blood pressure, heart rate, oxygen saturation, and end-tidal CO_2_, remained stable in all groups and anesthesia was uneventful in all patients. None of the patients had hypertension or tachycardia as a response to laryngoscopy. 

**Table 1:** Demographic data of the patients.

	Group F	Group C	Group M	P values
Sex (F/M)	10/10	12/8	10/10	0.765
ASA (I/II/III)	7/13/0	8/10/2	12/7/1	0.192
Mallampati (1/2/3)	6/10/4	8/11/1	5/13/2	0.536
Age (years)	49.9 ± 13.8	49.2 ± 11.5	45.6 ± 9.7	0.469
Height (cm)	168.0 ± 8.1	168.9 ± 8.3	167.0 ± 9.5	0.786
Weight (kg)	76.5 ± 18.0	74.8 ± 10.8	80.5 ± 8.8	0.199

The data are presented as n or mean ± standard deviation. Group M: Macintosh laryngoscope; Group F: LMA Fastrach; Group C: LMA CTrach.

Cervical alpha (α) angles in degrees at the C1/2 segment of the study patients are shown in Table 2. Baseline measurements were significantly different in Group M compared with the other groups (P = 0.004). According to the neutral baseline position, angulation of the C1/2 segment (α angle) decreased during intubation in Group F (P = 0.042) and in Group M (P = 0.001), whereas there was no significant difference in Group C (P = 0.159). The mean degree of the change in angulation compared with the preinduction baseline values at C1/2 1.2°, 1.1°, and 2.9° for Groups F, C, and M was not statistically significant.

**Table 2:** Alpha (α) angle measurements.

	Group F	Group C	Group M	P values
Control	74.5 ± 4.2	74.0 ± 5.4	69.6 ± 5.7	0.004‡
Laryngoscopy	73.3 ± 4.7*	72.9 ± 6.4	66.7 ± 6.2†	0.001‡

The data are presented as mean ± standard deviation. Group M: Macintosh laryngoscope; Group F: LMA Fastrach; Group C: LMA CTrach. * : P < 0.05, laryngoscopy versus control. † : P < 0.05, laryngoscopy versus control. ‡ : P < 0.05, Group M versus Group F and Group C.

Cervical beta (β) angles of the studied patients are displayed in Table 3 in degrees. At the C2/3 segment (β angle) Groups F and M similarly showed a significant increase (P = 0.001, P < 0.001, respectively) in cervical motion, while there was no difference in Group C during intubation compared with the neutral position. The mean change in β angle during intubation was prominent in Groups F and M, but in Group C the extension was statistically less (3.7°, 0.7°, and 7.1° for Groups F, C, and M).

**Table 3:** Beta (β) angle measurements.

	Group F	Group C	Group M	P values
Control	101.9 ± 8.4	101.9 ± 9.6	100.0 ± 6.3	0.702
Laryngoscopy	105.6 ± 9.7*	102.6 ± 8.3	107.1 ± 4.4†	0.107

The data are presented as mean ± standard deviation. Group M: Macintosh laryngoscope; Group F: LMA Fastrach; Group C: LMA CTrach. * : P < 0.05, laryngoscopy versus control. † : P < 0.05, laryngoscopy versus control.

The mean intubation time was 32 s in Group M, ranging from 10 to 120 s. It took the longest time to intubate patients in Group F and the shortest in Group M (P < 0.001) (Table 4). The mean duration of intubation was 98.8 and 61.0 s for the LMA Fastrach and LMA CTrach, respectively (P < 0.001). 

**Table 4:** The number of attempts, the duration, and the success rate of intubation.

	Group F	Group C	Group M
The number of attempts (1/2/3)	11/5/4§	15/5/0	18/2/0
Duration of intubation (s)	62.5 (30–300)*	40 (30–230)	20 (10–120)¶
The success rate	16 (%80)*	20 (%100)	20 (%100)

The data are presented as n, median ± minimum-maximum, or n (%). Group M: Macintosh laryngoscope; Group F: LMA Fastrach; Group C: LMA CTrach. §: P < 0.05, Group F versus Group M. * : P < 0.05, Group F versus Group C and Group M. ¶ : P < 0.05, Group M versus Group F and Group C.

The numbers of patients intubated in the first attempt were 18 with the Macintosh laryngoscope, 15 with LMA CTrach, and 11 with LMA Fastrach while two attempts were required for 2, 5, and 5 patients, respectively (Table 4). The number of attempts significantly differed in Group F compared to Group M (P = 0.016). It required more than two attempts and was successful after repositioning the LMA Fastrach in 4 patients. The rate of successful tracheal intubation was 80% with LMA Fastrach and 100% with LMA CTrach and Macintosh laryngoscopes. Group F had the statistically lowest success rate (P = 0.009).

## 4. Discussion 

The main result of this study is that the LMA CTrach significantly reduced extension compared to LMA Fastrach and Macintosh laryngoscopes at the C2/3 segment (β angle) without prolonging intubation time in patients undergoing elective C-spine surgery. According to our hypothesis, both LMA Fastrach and LMA CTrach would be associated with less cervical movement than Macintosh laryngoscopes, which was at least partially confirmed with LMA CTrach for the β angle. 

A study by Sawin et al. investigated the behavior of the intact C-spine during direct laryngoscopy with a Macintosh blade and proved the general assumption that a majority of cervical motions associated with laryngoscopy occur in the upper cervical region [4]. Subaxial segments (under C2) were displaced minimally. Thus, the present study was undertaken to quantify the motion of cervical segments 1/2 and 2/3. The times for radiography were chosen as once before induction and once during laryngoscopy immediately prior to insertion of the endotracheal tube or when the best view of the glottis was achieved as Hindman et al. reported maximal intubation biomechanics occurring at that stage [16]. 

A cadaver model of cervical instability found that supraglottic airways (LMA, ILMA) caused less or equal C-spine movement compared to the conventional laryngoscopes (Macintosh, McCoy) [9]. The authors therefore suggested that, due to the ease of training, supraglottic airways could be preferred in cervical trauma patients. Komatsu et al. tested ILMAs for controlling the airway in patients undergoing C-spine surgery who were wearing rigid cervical collars to simulate C-spine injury and found ILMAs a reasonable alternative for facilitating intubation [17]. 

Panjabi et al. defined the upper limits of the physiological motion as a rotation of over 20° in the sagittal plane [18]. The maximum cervical motion at C2/3 in our study was 15° for the Macintosh laryngoscope, 12.4° for LMA Fastrach, and 7.6° for LMA CTrach. In a video-fluoroscopic study, Sahin et al. observed a maximum movement of 18.5°, 16.7°, and 8.1° during direct laryngoscopy, intubation with ILMA, and fiberoptic laryngoscopy at C1/2 [6]. The maximum cervical motion produced with LMA Fastrach in both studies was close to the angle of the Macintosh laryngoscope. However, it was still lower than the instability limits argued by Panjabi et al. [18]. Even though the extension of the motion produced by LMA CTrach in the present study seems to be less than that produced by fiber optic laryngoscopy in Sahin’s study, it is actually difficult to compare the data of these similar studies. In the present study there was a significant difference in baseline angle measurements at C1/2. We do not recognize this as a study limitation because the initial position of the patient’s head cannot be standardized. However, the degree of cervical extension during laryngoscopy is important. The previous literature reported extension at C1/2 produced with ILMAs ranging from 1° to 5° and even as high as 7.4° [6,10–12]. Our result is in line with this range. Nevertheless, variability of the results depends on the heterogeneity introduced by methodological and population differences of the studies and experiences of the investigators. 

The ability to intubate a trachea under glottis visualization with LMA CTrach was reported with higher first attempt success rates compared with LMA Fastrach [14]. Liu et al. found a 98.9% first attempt success rate for LMA CTrach in 100 patients, while Baskett et al. showed a 79.8% success rate for LMA Fastrach with the experience of 500 cases [7,19]. Bilgin et al. demonstrated a first attempt success rate of intubation of 54% for ILMAs and 90% for C-Trach [13]. Our success rate, which is 75% with LMA CTrach and 55% with LMA Fastrach, is lower than those in the published literature. The diversity of these results is probably due to the methodological differences, the skills of the investigators, and the sample sizes of the mentioned studies. Nevertheless, in the current study, tracheal intubation was defined as successful only if the patient was intubated in two attempts at most with a device and the rate of successful tracheal intubation was 80% with LMA Fastrach, which statistically had the lowest rate.

Secondary spinal injury during airway management is not only a result of the mechanical disruption of the unstable segments, but hypoxia is also likely to cause harm [1,20]. Although both LMA CTrach and LMA Fastrach administrations may prolong intubation, they have established roles in difficult airway management since they do not interrupt ventilation [8,21]. Obviously, duration of intubation lasts longer with devices that require different maneuvers compared to the laryngoscopes. In the present study, as the most familiar device for anesthetists, the Macintosh laryngoscope intubation was fastest, while the duration of intubation was significantly longer with LMA Fastrach. These findings are in line with those of Bilgin et al., who reported significantly longer mean intubation time with an ILMA compared to C-Trach and McCoy [13]. Nevertheless, none of the patients in our study presented hypoxia throughout the intubation process. 

Randomizing the patients without considering their Mallampati scores and evaluating airway difficulties might be a limitation of our study. If the selection of the airway device depended on the possibility of a difficult airway, a selection bias would occur for LMA CTrach over blind intubation using LMA Fastrach. Nevertheless, Mallampati scores were identical between the groups. Another possible shortcoming of the study is that it was impossible to blind the investigator to the airway device and only an independent radiologist who measured the angles was unaware of the study group assignments. Several investigators may have had different skill levels and experiences, so only one investigator performed all laryngoscopies in order to minimize any confounding effects. The third limitation is that tracheal intubations were facilitated with muscle relaxants. Sawin et al. [4] suggested that muscle relaxation using neuromuscular blockade might reduce the need for cervical extension during laryngoscopy. However, even in injury settings muscle relaxants are used to ease the insertion of the endotracheal tube. Finally, the study conclusions may be limited since this project examined only two X-ray graphics instead of dynamic fluoroscopy.

In conclusion, airway management with minimal neck movement improves the success of the anesthetic management in C-spine surgery. Moreover, there are limited data that may help to understand C-spine kinetics of the patients with cervical pathologies, especially degenerative disorders requiring surgery. It is thus important to be familiar with the different intubation techniques. However, one should also be aware of their effects on cervical extension. We conclude that the reduced C-spine extension during intubation with LMA CTrach makes it a reasonable alternative compared to LMA Fastrach and Macintosh laryngoscopes in cervical surgery where cervical stability is a concern.
